# Nationwide Outcome of Tailored Surgery for Symptomatic Chronic Pancreatitis Based on Pancreatic Morphology: Validation of the International Guidelines

**DOI:** 10.1097/SLA.0000000000006176

**Published:** 2023-12-13

**Authors:** Charlotte L. Van Veldhuisen, Charlotte A. Leseman, Fleur E.M. De Rijk, Emmelie Nathalie Dekker, Martine J. Wellens, Nynke Michiels, Martijn W.J. Stommel, Christina Krikke, Hendrik Sijbrand Hofker, Jan Sven David Mieog, Stefan A. Bouwense, Casper H. Van Eijck, Bas Groot Koerkamp, Roel Haen, Marja A. Boermeester, Olivier R. Busch, Hjalmar C. Van Santvoort, Marc G. Besselink

**Affiliations:** * Department of Surgery, Amsterdam UMC, University of Amsterdam, Amsterdam, The Netherlands; † Amsterdam Gastroenterology Endocrinology Metabolism, Amsterdam, The Netherlands; ‡ Department of Research and Development, St. Antonius Hospital, Nieuwegein, The Netherlands; § Department of Gastroenterology and Hepatology, Erasmus University Medical Center, Rotterdam, The Netherlands; ∥ Department of Surgery, Erasmus MC Cancer Institute, Erasmus University Medical Center, Rotterdam, The Netherlands; ¶ Department of Gastroenterology and Hepatology, Isala Hospital, Zwolle, The Netherlands; # Department of Surgery, Leiden University Medical Centre, Leiden, The Netherlands; ** Department of Surgery, Radboud University Medical Center, Nijmegen, The Netherlands; †† Department of Surgery, University Medical Center Groningen, University of Groningen, Groningen, The Netherlands; ‡‡ Department of Surgery, Maastricht University Medical Center+, Maastricht, The Netherlands; §§ Department of Surgery, St. Antonius Hospital, Nieuwegein, The Netherlands; ∥∥ Department of Surgery, University Medical Center Utrecht, Regional Academic Cancer Center, Utrecht, The Netherlands

**Keywords:** surgical treatment, symptomatic chronic pancreatitis

## Abstract

**Objective:**

To determine the nationwide use and outcome of tailored surgical treatment for symptomatic chronic pancreatitis (CP) as advised by recent guidelines.

**Background:**

Randomized trials have shown that surgery is superior to endoscopy in patients with symptomatic CP, although endoscopy remains popular. Recent guidelines advice to “tailor surgery” is based on pancreatic morphology, meaning that the least extensive procedure should be selected based on pancreatic morphology. However, nationwide and multicenter studies on tailored surgery for symptomatic CP are lacking.

**Methods:**

Nationwide multicenter retrospective analysis of consecutive patients undergoing surgical treatment for symptomatic CP in all 7 Dutch university medical centers (2010–2020). Outcomes included volume trend, major complications, 90-day mortality, postoperative opioid use, and clinically relevant pain relief. Surgical treatment was tailored based on the size of the main pancreatic duct and pancreatic head (eg, surgical drainage for a dilated pancreatic duct and normal size pancreatic head).

**Results:**

Overall, 381 patients underwent surgery for CP: 127 surgical drainage procedures (33%; mostly extended lateral pancreaticojejunostomy), 129 duodenum-preserving pancreatic head resections (34%, mostly Frey), and 125 formal pancreatic resections (33%, mostly distal pancreatectomy). The annual surgical volume increased slightly (Pearson *r*=0.744). Mortality (90-d) occurred in 6 patients (2%) and was nonsignificantly lower after surgical drainage (0%, 3%, 2%; *P*=0.139). Major complications (12%, 24%, 26%; *P*=0.012), postoperative pancreatic fistula grade B/C (0%, 3%, 22%; *P*=0.038), surgical reintervention (4%, 16%, 12%; *P*=0.006), and endocrine insufficiency ( 14%, 21%, 43%; *P*<0.001) occurred less often after surgical drainage. After a median follow-up of 11 months (interquartile range: 3–23), good rates of clinically relevant pain relief ( 83%, 69%, 80%; *P*=0.082) were observed and 81% of opioid users had stopped using (83%, 78%, and 84%; *P*=0.496).

**Conclusions:**

The use of surgery for symptomatic CP increased over the study period. Drainage procedures were associated with the best safety profile and excellent functional outcome, highlighting the importance of tailoring surgery based on pancreatic morphology.

Chronic pancreatitis (CP) is a progressive inflammatory disease of the pancreas, often accompanied by severe pain and irreversible destruction of pancreatic parenchyma and associated loss of function.^1^ Pain in CP strongly impairs quality of life and is considered the main target for treatment.^2,3^ The randomized multicenter ESCAPE trial and previous randomized trials confirmed better outcomes with early surgery as compared to an endoscopy-first approach.^4–7^

Surgical treatment for symptomatic CP can be divided into surgical drainage procedures [eg, lateral pancreaticojejunostomy (LPJ)], duodenum-preserving pancreatic head resections (DPPHRs: eg, Frey, Beger, and Bern procedures), and formal pancreatic resections (eg, pancreatoduodenectomy, distal pancreatectomy, and total pancreatectomy—with or without islet autotransplantation).^8^ The choice of a specific surgical technique depends on pancreatic morphology, but may also be influenced by institutional policy and the surgeon’s preference.^4,6,7,9,10^

The recent multisociety international consensus guideline described a tailored approach to surgery for CP, based on the size of the pancreatic head and the diameter of the main pancreatic duct (ie, using cutoffs of 40 mm for the pancreatic head and 5 mm for the pancreatic duct).^11^ However, large, nationwide, and multicenter studies validating this guideline advice are lacking. At the same time, interventional endoscopy also increasingly plays a role in patients with symptomatic CP.^12^ The impact of these developments on the nationwide use of surgery in patients with symptomatic CP is also unclear.^13^

This nationwide study aimed to evaluate the use and outcome of surgical treatment for symptomatic CP, focusing on the outcome of tailored surgery based on pancreatic morphology using surgical drainage procedures, DPPHR, and formal pancreatic resections.

## METHODS

A nationwide retrospective multicenter study involving all 7 university medical centers in the Netherlands (ie, Amsterdam UMC, Erasmus MC Rotterdam, Leiden UMC, Maastricht UMC, Radboud UMC, Groningen UMC, and UMC Utrecht/St. Antonius hospital Nieuwegein). The study was coordinated by the Dutch Pancreatitis Study Group and conducted in accordance with the Strengthening the Reporting of Observational Studies in Epidemiology (STROBE) guidelines and approved by the medical ethics committee of Amsterdam UMC.^14^

### Study Population

All consecutive patients who underwent surgical treatment for symptomatic CP from January 1, 2010, to December 31, 2020, were identified from hospital records.^9^ Included were patients ≥18 years of age with symptomatic CP as the primary indication for surgery. The diagnosis of CP was made based on the clinical evaluation, clinical history, and radiologic imaging according to the M-ANNHEIM classification of CP.^15^ Surgical treatment included drainage procedures, DPPHR, and formal pancreatic resections. Excluded were patients undergoing total pancreatectomy with islet autotransplantation (ie, very few TPIAT procedures were done for symptomatic CP in the Netherlands during the study period), patients with pancreatic cancer in the final pathology rapport, and patients undergoing surgery for acute complications of CP.

### Endpoints

The study aimed to evaluate the trends in the use of surgical treatment of symptomatic CP, and to assess surgical outcomes, focusing on 90-day mortality and morbidity, postoperative opioid use, and clinically relevant pain relief, both overall and between the different types of surgical treatment. Other endpoints included indication and type of surgical approach, time from diagnosis to intervention, postoperative pancreatic function, reintervention, and treatment satisfaction during long-term follow-up. Pain relief was solely evaluated in patients who underwent surgical treatment with pain as the primary indication for surgery. The use of pain medication for CP after surgical treatment (ie, weak or strong opioids or other analgesics) was evaluated at the latest clinical follow-up.

### Definitions

Clinically relevant pain relief was considered as a decrease in pain during the follow-up period, defined as either complete (no more opioids) or partial (decreased use of opioids).^16,17^ Codeine and tramadol were considered weak opioids, whereas oxycodone, fentanyl, methadone, buprenorphine, diamorphine/heroin, dihydromorphine, and remifentanil were considered strong opioids. Other types of pain medication than opioids comprised paracetamol, nonsteroidal anti-inflammatory drugs, and neuropathic analgesics such as gabapentin. Endocrine and exocrine insufficiency was defined as the use of medication (ie, diabetes medication and enzyme replacement therapy respectively) or a fecal-elastase-1 test (<200 μg/g) for exocrine insufficiency.

Treatment satisfaction was scored as complete/partial versus no satisfaction, based on what patients reported during outpatient clinical follow-up. Among patients who reported pain relief after surgery, clinical outcome was divided into 3 categories: (1) continued pain relief: patients who reported pain relief both at the first and last follow-up; (2) discontinued pain relief: patients who initially reported pain relief, but later reported worsening of pain; (3) new-onset pain relief: patients who initially did not report pain relief but did report pain relief during follow-up.

A pancreatic head with a diameter of >40 mm was considered enlarged,^6^ as a main pancreatic duct of 5 mm was considered dilated.^18^ Pancreatic morphology of CP was described as normal or dilated main pancreatic duct, and enlarged or normal pancreatic head and other morphology (such as pseudocysts and groove pancreatitis). Etiology was reported according to the TIGAR-O classification.^19^ Indications to perform surgery were reported as either pain (defined as intractable pain or frequent flare-ups of pain) or complications due to CP (defined as common bile duct obstruction, duodenal or bowel obstruction, presence of pseudocysts, and other). Postoperative complications were reported using the Clavien-Dindo classification, in which a major complication is defined as grade 3 or higher.^20^. Postoperative pancreatic fistula (POPF) and postpancreatectomy hemorrhage were reported using the International Study Group for Pancreatic Surgery (ISGPS) classification.^21,22^ Only the clinically relevant grade B/C complications were included.

### Surgical Techniques

Surgical techniques for CP were classified into 3 categories: (1) surgical drainage procedures (ie, LPJ, which included extended LPJ^23^ and Partington-Rochelle, Puestow procedures; (2) DPPHR (ie, Frey, Beger, and Bern procedures), and (3) formal pancreatic resections (ie, pancreatoduodenectomy, distal pancreatectomy, and total pancreatectomy). Supplement 1 provides an overview of the different surgical techniques (Supplemental Digital Content 1, http://links.lww.com/SLA/E971), and Figure S1 illustrates the different surgical techniques (Supplemental Digital Content 1, http://links.lww.com/SLA/E971). No distinction was made between extended LPJ and “standard” LPJ for the purpose of the present study.^23^

### Data Collection

The coordinating and local researchers collected data in the participating centers. All patients who underwent surgical treatment for symptomatic CP in the participating centers in the period January 2010 to December 2020 were collected retrospectively from the electronic health records. An online electronic case report form (eCRF) in Castor (Castor EDC) was used.^10^

### Statistical Analyses

Descriptive statistics were used for patient, preoperative, and postoperative characteristics. Continuous data were summarized using mean±SD and ranges for normal distributed data, whereas non-normal distributed data were summarized using median with interquartile ranges (IQRs). Categorical data were presented as proportion in category. The study outcomes were compared per surgical technique category. Depending on the distribution of data, the one-way ANOVA or Kruskal-Wallis test was performed for continuous data. For categorical data, the χ^2^ test was used. In case of significant effects, cell statistics were examined to interpret the effect. *P* values were 2-tailed, and significance level was determined at *P*<0.05. IBM SPSS Statistics version 28 was used to perform the statistical analyses. Due to the retrospective design of the study, no sample size calculation was performed.

An additional logistic regression analysis was performed to investigate potential predictive factors for the incidence of clinically relevant pain relief. Based on previous literature, we defined the following preoperative characteristics as potential predictors: interval between diagnosis and surgery, the use and duration of opioids preoperatively, and smoking/alcohol use.^24^ Logistic regression analysis for association models was performed (with the forced entry of predictors). *P* values<0.05 were considered significant as entry-level in the model.

## RESULTS

### Baseline Characteristics

Overall, 386 patients had surgical treatment for symptomatic CP. From this group, 5 patients (1%) were excluded because pancreatic cancer was diagnosed in the resected specimen. The remaining 381 patients were included in this study. Baseline characteristics are shown in Table 1; 246 patients (65%) were male and the mean age was 52 years (range: 18–79). The most frequent etiology for CP was toxic in 216 patients (57%). The majority of patients were smokers (258 patients, 68%), with alcohol use at the time of surgery reported in 80 patients (21%). Before surgery, endocrine and exocrine pancreatic insufficiency had been diagnosed in 116 (31%) and 220 patients (58%), respectively. The majority of patients with pain as the primary indication for surgery, 241 out of 327 ( 74%, excluding patients who underwent gastro- or hepaticojejunostomy), used opioids preoperatively, mostly for 3 to 11 months (in 102 out of 241 patients, 31%). More than half of patients [211 patients (55%)] had undergone prior endoscopic treatment for CP. Trends in use and outcome of preoperative endoscopic treatment are provided in Supplement 2, Tables S1, S2, and S4, Supplemental Digital Content 1, http://links.lww.com/SLA/E971.

**TABLE 1:** Baseline Characteristics

	Total (n=381)
Age, years, mean (SD), range	51.9 (12), 18–79
Male, n (%)	246 (65)
Body mass index, mean (SD), range*	22.8 (3.8), 14.0–37.6
Etiology of CP, n (%)	
Toxic (eg, alcoholic)	216 (57)
Idiopathic	78 (21)
Genetic	10 (3)
Obstructive	30 (8)
Recurrent	27 (7)
Other^*^	20 (5)
Pancreatic function, n (%)†	
Endocrine insufficiency	116 (31)
Exocrine insufficiency‡	220 (58)
Use of opioid, no. of patients (%)§ ∥	241 (74)
Duration of opioid use, n (%)¶	
<3 mo	21 (9)
3–11 mo	102 (42)
>12 mo	86 (36)
Current alcohol use, n (%)#	80 (21)
Current tobacco use, n (%)**	258 (68)
Previous endoscopic procedure, n (%)	211 (55)

* Missing in 12 patients.

† Missing in 1 patients.

‡ Diagnosis based on the use of Pancreatic enzyme replacement therapy (PERT) or fecal-elastase-1 test <200 μg/L.

§ Missing in 2 patients.

∥ Number corresponding to patients with pain as indication for surgery (excluding hepaticojejunostomy/gastrojejunostomy procedures).

¶ Missing in 32 patients.

# Missing in 11 patients.

** Missing in 10 patients.

### Postoperative Outcome

**FIGURE 1:**
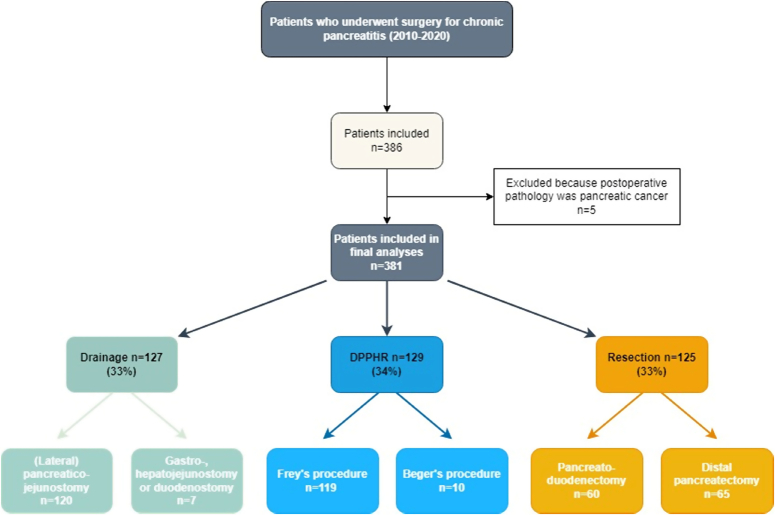
Surgical procedures.

Of the 381 patients, 127 patients (33%) underwent a surgical drainage procedure, 129 patients (34%) a DPPHR, and 125 patients (33%) a formal pancreatic resection. Within the surgical drainage group, 120 patients (94%) underwent (extended) LPJ and 7 patients (6%) underwent gastro- or hepaticojejunostomy. Within the DPPHR group, 119 patients (92%) underwent a Frey procedure and 10 patients (8%) underwent a Beger procedure. Within the formal pancreatic resection group, 60 of the 125 patients (48%) underwent a pancreatoduodenectomy, and the remaining 65 patients (52%) underwent a distal pancreatectomy (Fig. 1).

The pancreatic morphology of CP is shown in Table 2. A dilated main pancreatic duct (as a sole indication) was reported in 229 patients (61%), an enlarged pancreatic head (as a sole indication) in 18 patients (5%), and both a dilated main pancreatic duct and enlarged pancreatic head in 46 patients (12%). Other morphology was reported in 82 patients (22%), eg, multiple calcifications, (pseudo)cysts, groove pancreatitis, and pancreas divisum. The mean pancreatic duct size was 7.4 mm (SD: 3.0, range: 2.0–17.0), which differed significantly between the surgical groups (7.6 mm surgical drainage, 7.9 mm DPPHR, and 6.1 mm formal pancreatic resection; *P*=0.009). Pancreatic morphology was significantly associated with the type of surgical procedure; in the case of a dilated main pancreatic duct, formal pancreatic resections were less commonly performed compared to surgical drainage procedures and DPPHR (73% surgical drainage, 65% DPPHR, and 37% formal pancreatic resection; *P*<0.001). DPPHR procedures were performed more often if both the pancreatic duct and head were enlarged (10% surgical drainage, 18% DPPHR, and 7% formal pancreatic resection; *P*=0.027). Both in patients with an enlarged pancreatic head (2% surgical drainage, 5% DPPHR, and 8% formal pancreatic resection=0.036) and morphology described as other (15% surgical drainage, 13% DPPHR, and 47% formal pancreatic resection; *P*=<0.001), formal pancreatic resections were the most common.

**TABLE 2:** Pancreatic Morphology in 381 Patients Undergoing Surgical Treatment for Symptomatic Chronic Pancreatitis

	Total (n=381)	Surgical drainage (n=127)	DPPHR (n=129)	Pancreatic resection (n=125)	*P* *
Max. pancreatic duct size (mm)—mean (SD), range†	7.4 (3.0), 2.0–17.0	7.6 (2.8), 3.0–17.0	7.9 (3.3), 2.0–17.0	6.1 (2.6), 2.0–12.0	**0.009** β
Pancreatic morphology, n (%)‡					
Enlarged pancreatic head >4 cm	18 (5)	2 (2)	6 (5)	10 (8)	**0.036**
Dilated pancreatic duct >5 mm	229 (61)	93 (73)	83 (65)	45 (37)	**<0.001**
Enlarged pancreatic head and dilated pancreatic duct	46 (12)	13 (10)	23 (18)	9 (7)	**0.027**
Other	82 (22)	19 (15)	17 (13)	57 (47)	**<0.001**

Bold significances represent a difference between the 3 different groups (i.e. surgical drain age, DPPHR and resection).

Other morphology included multiple calcifications (37), (pseudo)cysts (15), groove pancreatitis (8), pancreas divisum (5), fluid collections (5), CBD and/or duodenal stenosis (4), normal pancreas (3), and unspecified (5).

All percentages reflect the total number of patients per subgroup including missing cases.

* Missing in 175 patients.

† Missing in 6 patients.

‡ χ^2^ or Fisher exact test was used for categorical variables.

β One-way ANOVA was used for normally distributed continuous data.

CBD indicates common bile duct.

The median time between diagnosis and surgery was 20 months (IQR: 7–51), which was 19 months (IQR: 6–39) in the surgical drainage group, 30 months (IQR: 10–62) in the DPPHR group, and 15 months (IQR: 5–54) in the formal pancreatic resection group, *P*=0.005.

Pain was the main indication for surgery, in 331 of the 381 patients (87%). In 22 patients (6%), other indications were reported, such as complications of severe malnutrition and recurring intra-abdominal pancreatic fluid collections or fistula.

Overall, 90-day mortality occurred in 6 patients (2%) and did not differ significantly between the groups (0%, 3%, and 2%; *P*=0.139). Major postoperative complications occurred in 78 patients (20%), less frequently after surgical drainage (12%, 24%, and 26%; *P*=0.012). The rate of POPF grade B/C was higher in the formal pancreatic resection group (0%, 3%, and 22%; *P*=0.038). The median length of hospital stay was 9 days [IQR: 7–12] and was shorter in the surgical drainage group [7 d (4–10) and 8 d (6–11) vs. 10 d (7–14); *P*<0.001].

In total, 39 patients (10%) needed a reintervention within 90 days after surgery, less often after surgical drainage (4%, 16%, and 12%; *P*=0.006). The surgical postoperative outcomes are presented in Table 3. Additional secondary outcomes are given in Table 4.

**TABLE 3:** Surgical Outcome

	Total (n=381)	Surgical drainage (n=127)	DPPHR (n=129)	Pancreatic resection (n=125)	*P* *
	Preoperative characteristics				
Interval between diagnosis and surgery, mo, median (IQR)†	20 (7–51)	19 (6–39)	30 (10–62)	15 (5–54)	**0.005** $
Indication for surgery, n (%)	6 (2)	0	4 (3)	2 (2)	0.139
Pain	331 (87)	114 (90)	112 (87)	105 (84)	0.399
Intractable	287 (75)	100 (79)	101 (78)	86 (69)	0.118
Frequent flare-ups	44 (12)	14 (11)	11 (9)	19 (15)	0.244
CBD obstruction	17 (5)	3 (2)	7 (5)	7 (6)	0.373
Pseudocysts	4 (1)	0	0	4 (1)	**0.013**
Duodenal obstruction	7 (2)	2 (2)	1 (1)	4 (3)	0.343
Other	22 (6)	8 (6)	9 (7)	5 (4)	0.568
	Postoperative outcome				
Mortality within 90 d, n (%)‡	6 (2)	0	4 (3)	2 (2)	0.139
Inpatient complication (Clavien-Dindo ≥grade 3), n (%)	78 (20)	15 (12)	31 (24)	32 (26)	**0.012**
Pancreatic fistula (grade B/C)∥	8 (2)	0	1 (3)	7 (22)	**0.038**
Postpancreatectomy hemorrhage (grade B/C)∥	5 (1)	2 (13)	3 (10)	0	0.139
Delayed gastric emptying	5 (1)	1 (7)	0	4 (13)	0.129
Chyle leakage	11 (3)	3 (20)	1 (3)	7 (22)	0.080
Wound infection	9 (2)	3 (20)	3 (10)	3 (9)	0.521
Pneumonia	7 (2)	1 (7)	3 (10)	3 (9)	0.940
Sepsis	14 (4)	5 (33)	5 (16)	4 (13)	0.210
Anastomotic leak	24 (6)	7 (47)	9 (29)	8 (25)	0.313
Bleeding	17 (5)	2 (13)	9 (29)	6 (19)	0.415
Intra-abdominal abscess	26 (7)	3 (20)	10 (32)	13 (41)	0.371
Other	34 (9)	4 (27)	12 (39)	18 (56)	0.127
Length of hospital stay, d, median (IQR)	9 (7–12)	7 (4–10)	8 (6–11)	10 (7–14)	**<0.001** $
Readmission within 90 d, n (%)‡	81 (21)	26 (21)	29 (23)	26 (21)	0.884
Reintervention within 90 d, n (%)§	39 (10)	5 (4)	19 (16)	15 (12)	**0.006**

Bold significances represent a difference between the 3 different groups (i.e. surgical drain age, DPPHR and resection).

All percentages reflect the total number of patients per subgroup including missing cases.

* χ^2^ or Fisher exact test was used for categorical variables.

† Missing in 14 patients.

‡ Missing in 2 patients.

§ Missing in 10 patients.

∥ According to ISGPS guidelines.

$ Kruskall-Wallis test was used for non-normal distributed data.

CBD indicates common bile duct.

**TABLE 4:** Secondary Outcomes

	Total (n=381)	Surgical drainage (n=127)	DPPHR (n=129)	Pancreatic resection (n=125)	*P* *
Duration of total follow-up, mo (IQR)†	11 (3–23)	11 (3–27)	12 (3–19)	11 (3–24)	0.868$
Clinically relevant pain relief, n (%)					
At first follow-up‡	216 (86)	77 (87)	73 (86)	66 (86)	0.987
At last follow-up§	201 (78)	80 (83)	56 (69)	65 (80)	0.082
Course in clinically relevant pain relief, n (%)∥					
Discontinued pain relief	18 (8)	5 (6)	6 (9)	7 (10)	0.716
Continued pain relief	154 (71)	61 (77)	41 (61)	52 (73)	0.092
New-onset pain relief	32 (15)	7 (9)	17 (25)	8 (11)	**0.012**
No pain relief	13 (6)	6 (8)	3 (5)	4 (6)	0.723
Patient-reported satisfaction, n (%)					
At first follow-up¶	199 (83)	70 (79)	66 (88)	63 (84)	0.273
At last follow-up#	170 (72)	64 (73)	53 (70)	53 (74)	0.857
Use of pain medication at the last follow-up, n (%)**					
Opioids	44 (19)	12 (17)	21 (22)	11 (16)	0.510
Weak	5 (2)	3 (4)	1 (1)	1 (1)	0.519
Strong	41 (17)	10 (14)	21 (22)	10 (14)	0.278
Other pain medication	36 (15)	9 (13)	18 (19)	9 (13)	0.413
New onset of pancreatic insufficiency at the last follow-up, n (%)††					
Endocrine insufficiency‡‡	67 (26)	12 (14)	16 (21)	39 (43)	**<0.001**
Exocrine insufficiency§§	71 (46)	22 (45)	17 (41)	32 (51)	0.571

Bold significances represent a difference between the 3 different groups (i.e. surgical drain age, DPPHR and resection).

All percentages reflect the total number of patients per subgroup including missing cases.

* χ^2^ test or Fisher exact was used for categorical variables.

† Missing in 27 patients.

‡ Missing in 76 patients.

§ Missing in 68 patients.

∥ Missing in 110 patients.

¶ Missing in 88 patients.

# Missing in 91 patients.

** Missing in 3 patients.

‡‡ Missing in 9 patients.

§§ Missing in 6 patients.

$ Kruskall-Wallis test was used for non-normal distributed data.

†† Numbers correspond to patients without preoperative pancreatic insufficiency.

### Pain Relief

The median follow-up after surgery was 11 months.^3–20^ Among the patients who underwent surgery because of pain (excluding the patients who underwent gastro- or hepaticojejunostomy), clinically relevant pain relief during the first follow-up was achieved in 216 out of 327 patients (86%), without differences between the groups (87%, 86%, 86%; *P*=0.987). During the last follow-up visit, clinically relevant pain relief was reported by 201 patients (78%) and also did not differ between the groups (83%, 69%, 80%; *P*=0.082, respectively). New-onset clinically relevant pain relief (not reported initially but only at later follow-up) was more often reported in the DPPHR group (9%, 25%, and 11%; *P*=0.012). Of the 241 out of 327 patients who preoperatively used opioids, and underwent surgery because of pain (ie, excluding the patients who underwent gastro- or hepaticojejunostomy), 81% had stopped at the last follow-up, which was similar between the groups (83%, 78%, and 84%; *P*=0.496). The use of other pain medication was reported in 36 patients (15%) without significant differences between the surgical groups (*P*=0.413).

### Treatment Satisfaction

Treatment satisfaction at the first follow-up visit was reported in 199 patients (83%), without differences between the groups (79%, 88%, 84%; *P*=0.273). During the last follow-up visit, treatment satisfaction was reported by 170 patients (72%) and also did not differ between the groups (73%, 70%, 74%; *P*=0.857, respectively).

### Pancreatic Insufficiency

Among the patients without preoperative pancreatic insufficiency, new-onset endocrine and exocrine pancreatic insufficiency was reported in 67 patients (26%) and 71 patients (46%), respectively. Endocrine insufficiency (14%, 21%, 43%; *P*<0.001) was more often seen after formal pancreatic resection, whereas exocrine insufficiency did not differ between the surgical groups (45%, 41%, 51%; *P*=0.571).

### Trends

**FIGURE 2:**
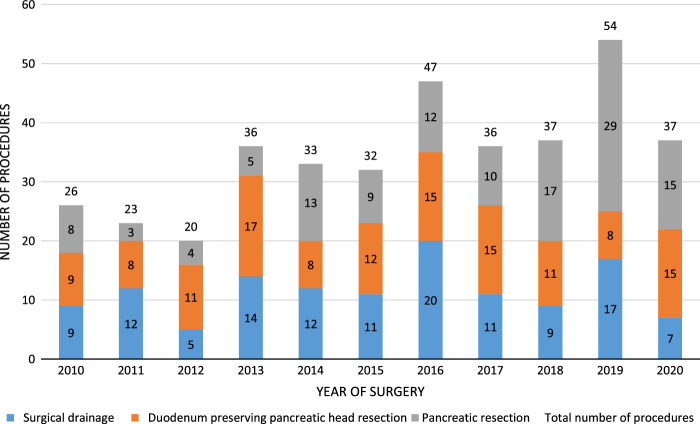
Nationwide trend in surgical treatment for chronic pancreatitis.

The volume of surgical procedures for symptomatic CP during the study period slightly increased (Fig. 2; Pearson *r*=0.744, *r* ^2^=0.554). When corrected for COVID-19, the linear association increased to *r*=0.816, *r* ^2^=0.667. The annual mean volume of surgery for symptomatic CP increased from 28 (SD: 6) in the period 2010–2015 to 42 in 2016–2020 (SD: 7, *P*=0.064).

### Pain Relief and Logistic Regression Analysis

The logistic regression analysis of the final prediction model for long-term clinically relevant pain relief after surgery for symptomatic CP is shown in Table 5. The model demonstrates that the preoperative absence of opioids and a short duration (<3 mo) of preoperative opioids is significantly associated with long-term pain relief [OR: 0.233 (95% CI: 0.092–0.589), *P*=0.002].

**TABLE 5:** Logistic Regression Analysis on Predictors for Long-term Clinical Pain Relief

Predictors	*B* (SE)	*P*	OR [95% CI]
Tobacco use	−0.580 (0.336)	0.086	0.560 [0.289–1.086]
Current alcohol use	0.684 (0.376)	0.069	1.982 [0.949–4.139]
Opioid use <3 mo*	−1.457 (0.473)	0.002	0.233 [0.092–0.589]
Opioid use 3–11 mo	−0.342 (0.280)	0.597	0.710 [0.200–2.519]
Opioid use >12 mo	0.043 (0.353)	0.902	1.044 [0.523–2.084]
Interval between diagnosis and surgery in months	0.005 (0.003)	0.079	1.005 [0.999–1.011]

Missing cases: 119; Nagelkerke *R* ^2^: 0.105; −2 Log likelihood 263.234, df=6.

* The compared variable is no opioid use preoperatively.

## DISCUSSION

This first nationwide and largest multicenter study to date in 381 consecutive patients found a slightly increased use of surgery for symptomatic CP. Surgical outcome was good, with the best safety profile for drainage surgery (90-d mortality 0%, POPF 0%, and major morbidity 12%). This highlights the importance of tailoring surgery to pancreatic morphology (ie, using the least extensive procedure required) especially since functional outcome was equally good after surgical drainage, DPPHR, and formal pancreatic resections with 86% clinically relevant pain relief. The different surgical strategies also performed equally well for patient satisfaction and the need for opioids post surgery. Altogether, these findings validate the advice for “tailored surgery” as given in the recent international guidelines.^11,25–27^

Our nationwide, multicenter findings are in line with series from single high-volume centers, which reported perioperative mortality of 1% (to 4.2% and postoperative opioid use of 17%).^25–27^ Furthermore, these studies also reported partial/complete pain relief in the majority of patients (65% and 86%). The current nationwide study also largely mirrors a previous large single-center Dutch study (1992–2006) with 0.9% mortality,^24^ highlighting the importance of centralization of pancreatic surgery as has been performed in the Netherlands.^28^ Currently, this is further investigated in the Dutch nationwide COMBO trial, which implements an evidence-based management algorithm for CP. In this study, patients who potentially require surgical intervention are discussed in a local multidisciplinary meeting and on indication separately in a nationwide expert panel.^29^ COMBO aims to further increase compliance with the European HaPanEU-guidelines, leading to improved quality of life for patients with symptomatic CP.^10,30^ Our results confirm the international consensus guidelines advice to tailor surgery for symptomatic CP based on the morphology of the main pancreatic duct, the pancreatic head, and the involvement of adjacent organs.^11^

Several studies have highlighted the importance of surgical timing in relation to clinical outcomes in patients with symptomatic CP. Our findings seem to confirm this hypothesis with regard to long-term pain relief. The time between first diagnosis and surgery in the current study was 20 months, which is relatively short. In the literature, this time period ranges from 27 to 48 months.^31–33^ Our current study period cannot yet evaluate the impact of the randomized ESCAPE trial as it was published in 2020. ESCAPE demonstrated that surgical treatment within 2 months of the start of strong opioids and within 6 months of the start of weak opioids was more effective than treating patients with a step-up approach starting with medical treatment and endoscopy.

In the current study, clinically relevant pain relief, evaluated after a median follow-up period of 11 months, was achieved in 201 patients (78%). Our findings are in line with previous studies, wherein pain relief in more than 70% of the patients was reported following LPJ and Frey procedures after a mean follow-up of 57 months (ie, Frey) and 67 months (ie, LPJ), respectively.^6,34^ Additional analyses on clinically relevant pain relief stratified per duration of follow-up are demonstrated in Supplement 2, Table S3, Supplemental Digital Content 1, http://links.lww.com/SLA/E971.

Interestingly, 72% of patients were satisfied with the outcome after surgery after a follow-up duration of 11 months. As the main indication for surgery was pain, perhaps even higher patient satisfaction rates could have been expected. This could be related to new-onset exocrine and endocrine insufficiency after surgery and progressing socioeconomic factors, which are known to have a significant impact on quality of life.^35,36^ Nevertheless, comparison of pain outcomes between studies is challenging, given the wide variety of methods that have been used to quantify pain relief after surgery.^37,38^ Therefore, more uniform measures and classification tools to describe pain assessment and pain relief should be implemented in future studies, which will allow more profound comparisons between studies.^39^

Over the years, the general perception of the treatment of CP has shifted toward a more proactive approach including early surgery.^7,33,40^ Despite an overall increasing trend in operations performed in this field, a decrease was seen during the last year. This is most likely explained by the COVID-19 pandemic, during which benign hepatobiliary surgery was frequently postponed.

There are some limitations that should be taken into account when interpreting the results of this study. First, this was a retrospective study over a period of 11 years, leading to incompleteness of surgical data and potential confounding factors. Second, subsequently, we did not prospectively evaluate numeric rating scale and Izbicki pain scores and pancreatic function was pragmatically evaluated based on information from the medical records.^41^ Third, data on the impact of surgery on quality of life are lacking, and therefore further research within this field is needed to perform a comprehensive analysis. Fourth, the duration of postoperative follow-up in the present study was relatively short.^26^ Fifth, a high number of patients (74%) used opioids before surgery, which is negatively associated with pain relief after surgery, confirmed by our regression analysis and in line with a previous study.^9^ Sixth, the patients in this cohort have specifically been selected to undergo surgery based on their symptoms and pancreatic morphology. Thereby, this cohort cannot directly be compared with patients treated endoscopically or conservatively. However, numerous previous randomized controlled trials have clearly shown the superiority of surgery over endoscopy in patients with morphine-dependent wide-duct CP.^6,7,13^ Conservative treatment is only very rarely used in these patients, if fit for treatment, since surgical treatment is so effective.

The main strength is the largest series of surgically treated patients with symptomatic CP to date which allowed for the assessment of trends over time and differences between surgical strategies for surgical and functional outcomes. The current series highlights the value of the recent international guidelines to tailor surgery on pancreatic morphology.^11^ This includes assessing the size of the pancreatic duct and the pancreatic head. Based on this the least invasive procedure should be selected. This approach will prevent morbidity while obtaining excellent functional outcomes.

## CONCLUSIONS

Surgery for symptomatic CP was safe with the best safety profile for surgical drainage procedures. In addition, the number of surgical procedures annually increased slightly over time. Pain relief was achieved in 78% of patients and remained mostly stable over time. Shorter use and duration of preoperative opioids at the time of surgery are associated with treatment success.

## Supplementary Material
