# Supplementary material for: Nationwide Outcome of Tailored Surgery for Symptomatic Chronic Pancreatitis Based on Pancreatic Morphology: Validation of the International Guidelines
Source: Ann Surg. 2023 Dec 13;281(4):655–63. doi: 10.1097/SLA.0000000000006176 (PMC11888824; doi:10.1097/SLA.0000000000006176)
Supplement: Supplementary file 1 [file sla-281-655-s001.docx]

# **SUPPLEMENTS**

**Supplementary 1** **Surgical techniques**

Below is a description of the surgical procedures performed in the present study. Figure 1 demonstrates the most common techniques.

**Drainage procedures**

Drainage procedures aim at decompression of ductal hypertension, which are often indicated in patients with an enlarged pancreatic duct and normal size pancreatic head.

*Lateral pancreaticojejunostomy*

In the lateral pancreaticojejunostomy (LPJ) according to Partington-Rochelle (also known as the modified Puestow procedure) the pancreatic duct is opened over its entire length to the left of the gastroduodenal artery, without resection of pancreatic tissue. Reconstruction is performed by a single side-to-side anastomosis with a Roux-en-Y loop on the opened pancreatic duct.

*Extended lateral pancreaticojejunostomy*

In the extended LPJ, the entire main pancreatic duct is opened over nearly the entire length which includes suture closing of the gastroduodenal arterial arcade. Subsequently, the risk of disease recurrence is minimized (Figure A). On both sides of the pancreatic duct (superior and inferior border), the gastroduodenal artery is identified and ligated. The pancreatic duct is opened from approximately 10 mm before the papilla of Vater to 10-20mm before the tip of the pancreas. Reconstruction is similar to the conventional LPJ, whereby the full length of the opened pancreatic duct is overlaid with a proximal Roux-en-Y loop.

**Formal pancreatic resection procedures**

Resection procedures for chronic pancreatitis (CP) are predominantly indicated in patients with an inflammatory mass in the pancreatic head or in patients with complications as a result of CP (such as pseudocysts).

*Pancreatoduodenectomy*

Pancreatoduodenectomy for CP is usually performed in patients with groove pancreatitis or morphological abnormalities in the pancreatic head. See Figure D.

*Distal pancreatectomy*

Distal pancreatectomy for CP is usually performed in patients in whom morphological changes are limited to the pancreatic body and tail or only the pancreatic tail.

*Total pancreatectomy*

Total pancreatectomy for CP is often seen as last resort treatment option, for example in patients with extensive fibrosis without endoscopic treatment options. During total pancreatectomy, the entire pancreas and gallbladder are removed, and in some centers, is followed by reimplantation of the patients’ own islet cells in the portal circulation to preserve some part of the islet cell function. Total pancreatectomy and auto islet transplantation (TPIAT) was not included in the current study.

**Duodenum preserving pancreatic head resections**

DPPHR procedures combine both drainage and resection techniques, which are often effective in patients with enlargement of the pancreatic head and dilation of the pancreatic duct.

*Beger*

During the Beger procedure, the pancreatic neck is resected above the portal vein, followed by a subtotal resection of the diseased parenchyma in the pancreatic head. Subsequently, a rim of pancreatic tissue remains along the duodenum. Two end-to-end pancreaticojejunostomies complete the reconstruction: one on the pancreas remnant and one on the rim of pancreatic tissue along the duodenum.

*Frey*

Instead of a transection at the pancreatic neck, the pancreatic head is cored out and the pancreatic neck is preserved, leaving a rim of pancreatic tissue along the duodenum. The gastroduodenal artery is identified and ligated below and above the pancreatic duct. Next, the main pancreatic duct is drained via a longitudinal incision over the entire length. Reconstruction is accomplished by a single side-to-side pancreaticojejunostomy similar to an extended lateral pancreatico-jejunostomy.

**Figure S1** Surgical treatment strategies for symptomatic chronic pancreatitis


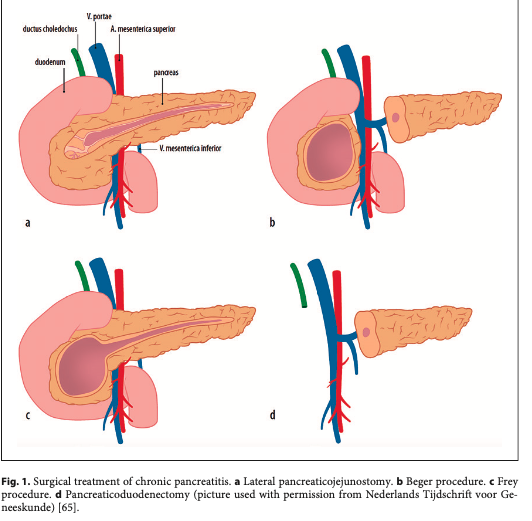


a. Extended lateral pancreaticojejunostomy , b. Beger procedure, c. Frey procedure, d. Pancreatoduodenectomy. Figure used with permission of Nederlands Tijdschrift Voor Geneeskunde (NTVG).

**Supplementary 2 Additional analyses**

**Table S1** Pre-operative endoscopic procedures for chronic pancreatitis

|  | **Total** (n=381) | **Surgical drainage** (n=127) | **DPPHR**  (n=129) | **Pancreatic resection** (n=125) | ***p*-value^a^** |
| --- | --- | --- | --- | --- | --- |
| **Prior endoscopic procedure,** no. of patients (%)^1^ | 211 (55) | 70 (55) | 84 (65) | 55 (45) | **0.005** |
| **Number of endoscopic procedures,** mean (SD), range^2^ | 3.3 (2.5), 0-20 | 3.1 (2.9), 1-20 | 3.5 (2.3), 1-14 | 3.3 (2.4), 0-12 | 0.739^$^ |
| **Stone removal,** no. (%)^1^ | 43 (11) | 15 (12) | 21 (16) | 7 (6) | **0.026** |
| **Stent placement,** no. (%)^1^ | 151 (40) | 41 (32) | 65 (50) | 45 (37) | **0.008** |
| **Location stent,** no. (%)^3^ | 150 (39) |  |  |  |  |
| Pancreatic duct | 55 (37) | 20 (49) | 21 (33) | 14 (31) | 0.451 |
| Common bile duct | 39 (26) | 5 (12) | 31 (33) | 13 (29) | **0.003** |
| Pancreatic duct and common bile duct | 14 (9) | 3 (7) | 4 (6) | 7 (16) | 0.359 |
| Unknown or removed at time of surgery | 42 (28) | 13 (32) | 18 (28) | 11 (24) | 0.399 |
| ^1^ missing in 2 patients, ^2^ missing in 7 patients, ^3^ missing in 1 patient.  ^a^ Chi-square or Fishers exact test was used for categorical variables.^$^ Oneway-ANOVA was used for normal distributed data. All percentages are reflecting the total number of patients per subgroup including missing cases. | | | | | |

**Table S2** Trends in endoscopic procedures

Multiple linear regression was used to test if the year of surgery predicted the number of patients who underwent endoscopic intervention before the surgical procedure. Table S2.5 demonstrates the results of a linear regression analysis with the number of patients who had an endoscopic intervention prior to surgery as dependent variable*.* A significant linear correlation was found between the year of surgery, the number of surgical procedures per year and the number of patients that underwent endoscopic intervention prior to surgery (adjusted R square = 0.903, F(2) = 47.074, p<0.001). However, on individual level, the year in which the surgical procedure was performed, was not significantly associated with the number of patients that underwent endoscopic intervention preoperatively.

| **Multivariate linear regression analysis*** | | |
| --- | --- | --- |
| **Variable** | **Beta** | **p-value^α^** |
| Year of surgery | -0.223 | 0.437 |
| Number of surgical procedures per year | 0.650 | <**0.001** |
| *Predictors for the number of patients with preoperative endoscopic intervention. ^α^ T-test was used to test significance.  Adjusted R square = 0.903, df 2 | | |

**Table S3** Clinically relevant pain relief per duration of follow-up

|  | | | | | |
| --- | --- | --- | --- | --- | --- |
|  | **< 3 months** (n=85) | **4-11 months**  (n=74) | **12-23 months  (**n=71) | **≥ 24 months**  (=74) | ***p*-value^a^** |
|  |  | | | | |
| **Clinically relevant pain relief,** no.(%) |  |  |  |  |  |
| At first follow-up^1^ | 56 (82) | 58 (92) | 48 (87) | 49 (82) | 0.305 |
| At last follow-up^2^ | 52 (84) | 56 (85) | 51 (79) | 42 (62) | **0.005** |
| **Course in clinically relevant pain relief**, no. (%)^3^ |  |  |  |  |  |
| Discontinued | 4 (8) | 3 (5) | 5 (10) | 6 (11) | 0.753 |
| Continued | 41 (77) | 46 (82) | 34 (67) | 32 (56) | **0.012** |
| New onset | 3 (6) | 5 (9) | 10 (20) | 15 (26) | **0.009** |
| No pain relief | 5 (9) | 2 (4) | 2 (4) | 4 (7) | 0.534 |
| **Patient reported satisfaction,** no. (%) |  |  |  |  |  |
| At first follow-up^4^ | 60 (85) | 53 (90) | 41 (84) | 40 (71) | 0.065 |
| At last follow-up^5^ | 49 (85) | 48 (79) | 38 (68) | 34 (54) | **0.001** |
| **Use of pain medication at last follow-up,** no. (%) |  |  |  |  |  |
| Weak | 1 (1) | 4 (4) | 1 (2) | 3 (4) | 0.465 |
| Strong | 13 (13) | 16 (16) | 8 (12) | 10 (13) | 0.910 |
| Other pain medication | 10 (10) | 17 (17) | 9 (14) | 9 (12) | 0.534 |
| Includes only patients who had pain as primary indication for surgery. ^1^ missing in 85 patients, ^2^ missing in 70 patients, ^3^ missing in 114 patients, ^4^ missing in 96 patients, ^5^ missing in 93 patients. ^a^ Chi-square or Fishers exact test was used for categorical variables. All percentages are reflecting the total number of patients per subgroup including missing cases. | | | | | |

**Table S4** Clinically relevant pain relief compared between endoscopically treated and non-endoscopically treated patients

|  |  | |  |
| --- | --- | --- | --- |
|  | **No preoperative endoscopic intervention**  (n=39) | **Preoperative endoscopic intervention**  (n=45) | **p-value^a^** |
| **Clinically relevant pain relief**, no. (%) |  |  |  |
| At first follow-up^1^ | 38 (97) | 26 (57) | 0.791 |
| At last follow-up^2^ | 35 (90) | 25 (56) | 0.434 |
| **Use of opioids at last follow-up,** no. (%)^3^ | 1 (3) | 2 (4) | 0.591 |
| Includes only patients who had pain as primary indication for surgery and did not use opioids preoperatively. ^1^ missing in 18 patients. ^2^ missing in 17 patients. ^3^ missing in 1 patient. ^a^ Chi-square or Fishers exact test was used for categorical variables. All percentages are reflecting the total number of patients per subgroup including missing cases. | | | |
